# Regulation of lnc-TLCD2-1 on Radiation Sensitivity of Colorectal Cancer and Comprehensive Analysis of Its Mechanism

**DOI:** 10.3389/fonc.2021.714159

**Published:** 2021-07-15

**Authors:** Qifeng Yu, Wei Zhang, Xin Zhou, Wenqi Shen, Chungen Xing, Xiaodong Yang

**Affiliations:** ^1^ Department of Gastrointestinal Surgery, The Second Affiliated Hospital of Soochow University, Suzhou, China; ^2^ Department of Radiology, The Second Affiliated Hospital of Soochow University, Suzhou, China

**Keywords:** lnc-TLCD2-1, radiation sensitivity, colorectal cancer, prognosis, biomarker

## Abstract

As is well known that colorectal cancer is the third most common cancer in the world, and radiation treatment plays a vital role in colorectal cancer therapy, but radiation resistance is a significant problem in the treatment of colorectal cancer. As an important member of the non-coding RNA family, long non-coding RNAs (lncRNAs) have been found that it plays a role in the occurrence and progression of colorectal cancer in recent years. However, little is known about the effect of lncRNA on colorectal cancer sensitivity to radiotherapy. We found that lnc-TLCD2-1 was significantly differentially expressed in radiation-tolerant CCL244 cell lines and radiation-sensitive HCT116 cell lines, suggesting that lnc-TLCD2-1 may regulate the radiosensitivity of colorectal cancer, and the relevant underlying mechanism was investigated. Cell clone formation assay, flow cytometry, and cell counting kit 8 (CCK8) were used to detect radiation sensitivity, apoptosis, and proliferation of colorectal cancer cells, respectively; Quantitative real-time PCR and western blot were used to detect the expression of genes; the direct interaction between lnc-TLCD2-1 and hsa-miR-193a-5p was verified by dual luciferase reporter assays; GEPIA, Starbase, TIMER and DAVID were used to complete expression of lnc-TLCD2-1, miR-193a-5p,YY1 and NF-кB-P65 in colorectal cancer, correlation, immune cell infiltration, GO and KEGG enrichment analysis. Clinical prognostic analysis data were obtained from GSE17536 dataset. After radiotherapy for HCT116, the expression of lnc-TLCD2-1 was increased, and the expression of hsa-miR-193a-5p was significantly decreased, while that of CCL244 was the opposite, and the change range of lnc-TLCD2-1 was relatively small. HCT116 with overexpression of lnc-TLCD2-1 after radiation treatment, the number of cell colonies significantly increased, and cell apoptosis decreased compared with the negative control group. The cell colonies and apoptosis of CCL244 with disturbed expression of lnc-TLCD2-1 were opposite to those of HCT116. Lnc-TLCD2-1 can regulate the expression of YY1/NF-кB-P65 by targeting miR-193a-5p. Lnc-TLCD2-1 can promote the proliferation of colorectal cancer. High expression of lnc-TLCD2-1 independently predicted a shorter survival. Lnc-TLCD2-1 is associated with radiation resistance and short survival in colorectal cancer patients. In addition, Lnc-TLCD2-1 can promote the proliferation of colorectal cancer. Our study provides a scientific basis for targeting lnc-TLCD2-1 in colorectal cancer radiation resistance interventions and selection of prognostic biomarker.

## Introduction

Globally, colorectal cancer (CRC) is one of the most common malignant cancers (1), it is second only to lung cancer, liver cancer and stomach cancer. CRC causes about 0.7 million deaths a year, making it the fourth leading cause of cancer death worldwide (2, 3). At present, CRC is the fastest growing cancer in Chinese society (4), which has caused great economic pressure on people’s lives and social development. Radiation therapy can improve the prognosis of patients with CRC, especially for locally advanced CRC patients has more important significance, Unfortunately, one third of patients with CRC show low sensitivity or complete resistance to radiation therapy (5). As an important member of the non-coding RNA family, long non-coding RNAs (lncRNAs) are RNA molecules over 200 nucleotides in length and lack of protein-coding capacity (6). The effect of lncRNA on proliferation, apoptosis, invasion and migration of tumor was mainly studied in the past (7). Currently, there are few reports on the regulation of lncRNAs on radiotherapy sensitivity of CRC. As for lnc-TLCD2-1, its regulation of tumor radiotherapy sensitivity has not been reported so far (The sequence of lnc-TLCD2-1 was provided in **Supplementary Data Sheet 1**).

There has been a lot of evidence to prove the interaction between lncRNAs and miRNAs, and this is often a negative regulatory relationship (8–10). Yin Yang 1 (YY1) is an evolutionally-conserved C2H2 zinc finger type multidomain transcription factor. Its role in promoting or inhibiting tumor growth is still controversial, and its regulatory role may depend on different cancers and different cell types (11). YY1 can bind to NF-кB-p65 to form a complex and regulate the transcription of genes (12–14). The radiation tolerance of ESCC TE-1 cells is related to the high expression of YY1, which can inhibit the proliferation of esophageal squamous cell carcinoma cells (15). Recent studies have shown that YY1 expression can promote the proliferation of colorectal cancer (16). Meanwhile, in human endometrioid endometrial adenocarcinoma, hsa-miR-193a-5p act directly on YY1 to inhibit its transcription and translation, thereby inhibiting the proliferation and migration of cancer cells (17).So far, there have been no reports on whether lnc-TLCD2-1 can interact with hsa-miR-193a-5p in CRC to regulate YY1/NF-кB-p65 and affect the sensitivity of CRC to radiotherapy.

To explore the influence of lnc-TLCD2-1 on the sensitivity of CRC to radiotherapy and its related mechanism is of far-reaching significance for clinical treatment of CRC and the prognosis of patients.

## Materials and Methods

### Tissue Specimens

Tumors and paracancerous tissues from 10 patients with CRC were obtained from the Second Affiliated Hospital of Soochow University and stored in liquid nitrogen immediately until use. Each colorectal cancer patient was diagnosed histopathologically by two independent pathologists, and clinicopathological factors in colorectal cancer patients included gender, age, tumor size, TNM stage, lymph node metastasis, and distant metastasis. All CRC patients signed written informed consent, and this work was approved by the research ethics committee of the Second Affiliated Hospital of Soochow University.

### Cell Culture

Human colon cancer cell lines (HCT116 and CCL244) were purchased from American Type Culture Collection (ATCC, Manassas, VA, USA). These cells were cultured in Dulbecco’s modified Eagle’s medium (DMEM, Thermo Fisher Scientific, Waltham, MA, USA) containing 10% fetal bovine serum (FBS, Gibco) and 1% Penicillin-Streptomycin (10,000 IU Pen/ml, 10,000 ug Strep/ml, MP Biomedicals, USA) under humidified atmosphere containing 5% CO_2_ at 37°C.

### Cell Transfection

Small interfering RNA (silnc-TLCD2-1) and overexpressed vector (veclnc-TLCD2-1) targeting lnc-TLCD2-1, miRNA mimic and inhibitor (miR-193a-5p mimic and inhibitor miR-193a-5p) targeting hsa-miR-193a-5p, and the corresponding negative controls (including si-NC, vec-NC, mimic NC and inhibitor NC) were all synthesized by Genepharma (Shanghai, China). We transfected the above oligonucleotides or vectors into CCL244 or HCT116 by lipofectamine 2000 reagent (Invitrogen, USA) in strict accordance with the instructions.

### Cell Clone Formation Assay

Mix 1.2% agarose and 2×DMEM medium (containing 2% Penicillin-Streptomycin and 20% FBS) at 1:1, add 1.0 mL to each well of the six-well plate, and let it stand at room temperature until set. Mix 0.7% agarose and 2×DMEM medium (containing 2% Penicillin-Streptomycin and 20% FBS) at 1:1, the cells in logarithmic growth phase were digested with 0.25% trypsin and diluted to 10000/ml. The cell suspension was added to the low concentration agarose solution mentioned above to achieve a final cell concentration of 1000/ml. Next, add 1ml of the mixture to each well in a six-well dish. After the upper agarose solidified, culture at 5%CO2 at 37°C for 2 weeks(add 200ul DMEM (containing 1% Penicillin-Streptomycin and 10% FBS) every 2 days and keep it moist). Each well was stained with 0.005% crystal violet (1ml) for 1h, and the number of communities was calculated with Image-Pro Plus6.0 after photographing.

### Quantitative Real-Time Polymerase ChainReaction (qRT -PCR)

We used Trizol Reagent (Invitrogen, USA) to extract the total RNA of cells strictly according to protocal, and the synthesis of complementary DNA (cDNA) was made by using Hifair^®^ II 1st Strand cDNA Synthesis Kit (YEASEN, China) done. As for miRNA, cDNA synthesis was performed using the Takara RNA PCR Kit (Takara, China). Subsequently, quantitative real-time PCR was performed to detect the gene expression, using Hieff^®^ qPCR SYBR Green Master Mix (Low Rox) (YEASEN, China). Relative expression using glyceraldehyde-3-phosphate dehydrogenase (GAPDH)or U6 calibration as a reference gene, then use “2 ^- Δ Δ CT^” method to calculate. The primer sequences used are as follows : GAPDH 5ʹ- GAAGTATGACAACAGCCTCAAGA-3ʹ(forward),5ʹ-TCTTCTGGGTGGCAGTGATG-3ʹ (reverse);U6 5ʹ-AAAATTTCTCACGCCGGTATTC-3ʹ(forward),5ʹ-CCTGCAGACCGTTCGTCAA-3ʹ (reverse); lnc-TLCD2-1 5ʹ- ATTAGAGACACTGGCTGGATT-3ʹ(forward), 5ʹ- AGTAAGAGCAGAAGGATGACT-3ʹ (reverse);NF-кB-P65 5ʹ-TGGAGCAGGCTATCAGTCA-3ʹ(forward), 5ʹ-TCGGTTCACTCGGCAGAT-3ʹ (reverse); YY1 5ʹ-AAGAGCGGCAAGAAGAGTTA-3ʹ(forward), 5ʹ-GAATAATCAGGAGGTGAGTTCTC-3ʹ (reverse); hsa-miR-193a-5p 5ʹ-ACGCTGGGTCTTTGCGG -3ʹ(forward); 5ʹ-TATGGTTGTTCACGACTCCTTCAC -3ʹ (reverse).

### Western Blot

The proteins were extracted from the transfected cells using RIPA lysis buffers (Beyotime, Shanghai, China) and then quantified using bicinchoninic acid (BCA) following the manufacturer’s instructions. An equal amount of the extract was separated by electrophoresis with 10% sodium dodecylsulfate polyacrylamide gel and transferred to a nitocellulose membrane. After blocking with 3% non-fat milk for 1 hour, the membrane was incubated with primary antibodies against YY1, NF-кB-P65 and GAPDH and then interacted with secondary antibodies conjugated with horseradish peroxidase. Finally, the immune response signals were observed by enhanced chemiluminescence.

### Cell Counting Kit-8 (CCK-8) Assay

Proliferative activity of colon cancer cell lines was measured using CCK-8(Beyotime, Shanghai, China) in a 96-well plate according to standard protocols.HCT116 transfected with vector plasmid and CCL244 transfected with small interfering RNA were transplanted into 96-well plates (1000cells/well), and the cells were incubated at 37°C in 5%CO_2_ atmosphere for 72 h. After 72 hours, add 10 microliters of CCK-8 reagent to each well, shake gently and mix well, continue to incubate at 37°C in 5%CO2 for 2 hours. The absorbance at 450 nm was measured with a microplate reader (Thermo Fisher Scientific, Waltham, MA, USA) to determine cell viability. Cell viability * (%) =[A(treatment)-A (empty culture)]/[A (Negative control)-A (empty culture)] ×100%.

### Flow Cytometry Assay

After the cells were transfected and irradiated, apoptosis was detected using the Annexin V-FITC/PI Apoptosis Assay Kit (BD Biosciences, San Jose, CA, USA) according to the standard protocol. The cells were digested with 0.25% trypsin, and the concentration of the cells to be tested was adjusted to about 5×10^5/ml. 1ml cells were taken, centrifuged at 1000rpm at 4°C for 5 min, and supernatant was discarded. Add 1ml of pre-cooled PBS and gently shake the cells to suspend. The Binding Buffer was diluted 1:3 with sterile deionized water (4 ml Binding Buffer +12 ml sterile deionized water). The cells were resuspend with 250 μl Binding Buffer and the concentration was adjusted to 1×106/ml. Take 100 μl cell suspension into 5 mL flow tube, add 5 μl Annexin V/FITC and 10 μl 20 μg/mL PI solution. Mix well and incubate at room temperature in dark for 15 minutes. Add 400 μl PBS to the reaction tube and analyze by flow cytometry. The blank group and treatment group were set up. The blank control group was negative control without dye treatment, and the treatment group was Annexin V-FITC and PI double staining. Annexin V-FITC and PI positive limits were set in the experimental group according to the negative control group.

### Dual-Luciferase Reporter Assay

Partial wild-type (wt) or mutated (mut) DNA sequences of hsa-miR193a-5p binding sites in lnc-TLCD2-1 3’UTR were amplified by PCR. Then, it was cloned into pmirGLO vectors (Promega, Shanghai, China) to obtain lnc-TLCD2-1-wt and lnc-TLCD2-1-mut reporter plasmids. Subsequently, HCT116 and CCL244 were co-transfected with the constructed reporter plasmid and miR-193a-5p mimic or inhibitor, and negative control was set in each group. Cells were collected at 48 h transfection, Dual Luciferase Reporter Gene Assay Kit (YEASEN, Shanghai, China) was used to detect luciferase activity.

### PrognoScan

PrognoScan (http://gibk21.bse.kyutech.ac.jp/PrognoScan/index.html) is able to assess the biological relationship between gene expression and clinical prognosis using published cancer gene sequencing data sets (18). It can be used to evaluate potential tumor markers and therapeutic targets. In our study, the survival outcome analysis of the public data set GSE17536 (19) was performed with the help of PrognoScan.

### GEPIA

GEPIA (Gene Expression Profiling Interactive Analysis, http://gepia.cancer-pku.cn/index.html) is developed by Zefang Tang, Chenwei Li and Boxi Kang of Zhang Lab, Peking University. It has a wide range of functions, including tumor/normal differential expression analysis, survival prognostic analysis, similar gene detection, correlation analysis and dimensionality reduction analysis based on cancer type or pathological stage (20). Here, we used GEPIA to obtain the top 100 genes most similar to YY1 in colorectal cancer.

### DAVID6.8

DAVID (http://david.abcc.ncifcrf.gov/) recently updated is in March 2017, it is an online bioinformatics resources, aiming at the function of a large number of genes or proteins, annotations, it is the full name of The Database for the Annotation, the Visualization and Integrated Discovery (21 **)**. We used David to carry out GO and KEGG enrichment analysis on 100 genes including YY1 and its similar genes. GO analysis included three aspects: molecular function, biological process and cellular component; KEGG(Kyoto Encyclopedia of Genes and Genomes) is an enrichment analysis of gene molecular signaling pathways.

### TIMER2.0

The full name of the TIMER2.0 (https://cistrome.shinyapps.io/timer/) database is Tumor Immune Estimation Resource, it can be used to query tumor immune, clinical and genomic characteristics (22). In this study, we calculated the correlation between the abundance of 6 kinds of immune cells (B cells, CD4+ T cells, CD8+ T cells, neutrophils, macrophages and dendritic cells) and the expression level of YY1 in colorectal cancer and rectal cancer by using TIMER.

### Statistical Analysis

Statistical data of the three repeated experiments were analyzed by GraphPad Prism 8 software (GraphPad Software Inc., San Diego, CA, USA). One-way ANOVA, two-way ANOVA and Student’s T-test were used to compare the differences between groups. The correlation between lnc-TLCD2-1 and miR-193a-5p was analyzed using Pearson Pearson’s chi-square test. P < 0.05 indicated that the difference was statistically significant.

## Results

### The Expression of lnc-TLCD2-1 and hsa-miR-193a-5p in CRC Cells Was Changed After Irradiation

**Figure 1:**
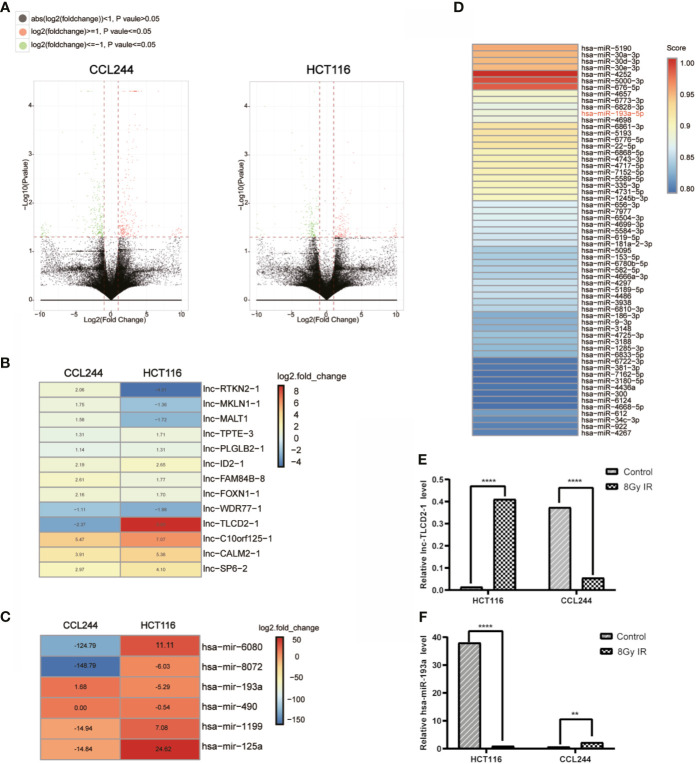
Difference in RNA expression between CCL244 and HCT116 after irradiation. **(A)** LncRNAs differentially expressed in CCL244 and HCT116 after 8 Gy radiation exposure. (log2(foldchange)>=1 or log2(foldchange)<=-1, and p value<=0.05 were considered a statistically significant difference). **(B)** CCL244 and HCT116 co-exist differentially expressed lncRNAs. **(C)** Six miRNAs with differences were detected. **(D)** Starbase database predicted the interaction of lnc-TLCD2-1 with miRNA (Score>=0.8). **(E)** The expression of lnc-TLCD2-1 was significantly changed after radiation. **(F)** MiR-193a-5p was significantly changed after radiation. (P<=0.05 means statistically significant, **p < 0.01, ****p < 0.0001.)

We already reported before in HT29 and LOVO, CACO2, CCL244, HCT116, SW480, HCE8693 these seven kinds of colorectal cancer cell lines, CCL244 for the most resistance to radiation, and HCT116 is the most sensitive to radiation (23). To identify potential lncRNAs that regulate the radiosensitivity of CRC cells, we first screened for the aberrantly expressed lncRNAs. Analysis of gene expression levels of CCL244 without and after radiation revealed 905 RNAs with significant differences (log2foldchange>=1, p value<=0.05), meanwhile, there were 606 differentially expressed RNAs inHCT116 (**Figure 1A**, **Supplemental Tables 1** and **2**). **Figure 1B** demonstrated 13 lncRNAs shared by the two types of cells with significantly different expressions. Obviously, among the 13 lncRNAs, lnc-TLCD2-1 showed the largest increase after HCT116 was irradiated. Meanwhile, this molecule was down-regulated after CCL244 was irradiated, but the decrease was not very large. Differentially expressed miRNAs are shown in **Figure 1C**. Starbase database predicted the interaction of lnc-TLCD2-1 with miRNA and was surprised to find that hsa-miR-193a-5p might be the target of lnc-TLCD2-1 (**Figure 1D**), this also confirmed the sequencing results, which showed that hsa-miR-193a significantly decreased after HCT116 was irradiated, while that of CCL244 was reversed (**Figures 1E, F**).

### The Expression of lnc-TLCD2-1 Is Generally Low in CRC, and the High Expression of It Predicts a Short Survival

**Figure 2:**
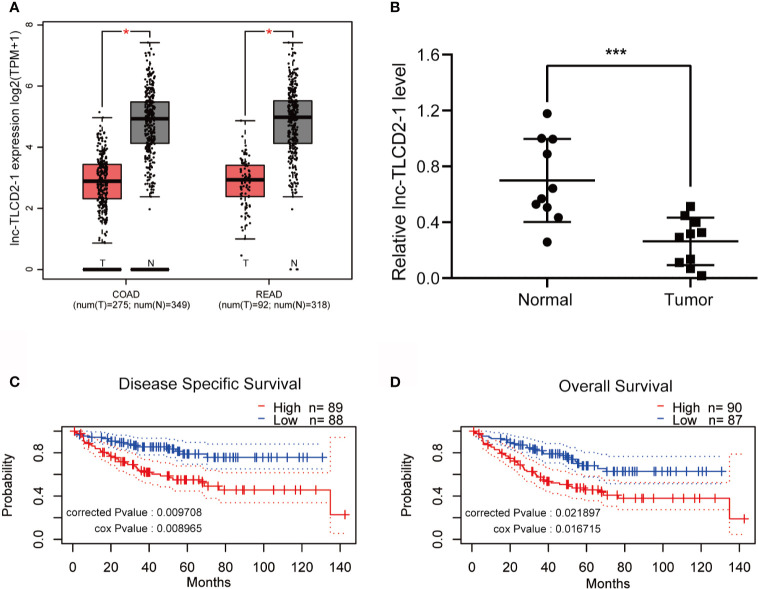
The expression of lnc-TLCD2-1 is generally low in CRC, and the high expression of it predicts a short survival. **(A)** TCGA database revealed low expression of lnc-TLCD2-1 in colorectal cancer (COAD stands for colon cancer, READ stands for rectal cancer). **(B)** The low expression of lnc-TLCD2-1 in CRC tissues were verified in 10 paired samples by qRT-PCR. **(C, D)** Disease Specific Survival and Overall survival analysis results were obtained from GSE17536 dataset, high expression of lnc-TLCD2-1 predicts a short survival. (P<=0.05 means statistically significant, *p < 0.05, ***p < 0.001.)

We used GEPIA to search for differences in the expression of lnc-TLCD2-1 between CRC and normal colorectal tissue. Compared with normal colorectal tissues, the expression of lnc-TLCD2-1 was decreased in CRC tissues (**Figure 2A**). In addition, lnc-TLCD2-1 was significantly low-expressed in 10 CRC tissues compared with matched normal tissues by qRT-PCR (**Figure 2B**), this further confirmed the analysis results of the database. Next, we studied the influence of the expression of lnc-TLCD2-1 on the survival and prognosis of CRC patients. The disease-specific survival (DSS) of 88 patients with low expression level was significantly higher than that of 89 patients with high expression level (corrected P value=0.009708, cox P value=0.008965) (**Figure 2C**). And the Overall Survival (OS) of 87 patients with low expression was significantly higher than that of 90 patients with high expression (corrected P value=0.021897, cox P value=0.016715) (**Figure 2D**). These results were obtained by our meta-analysis of the prognostic value of genes in the public data set GSE17536 using PrognoScan. This suggests that although the majority of lnc-TLCD2-1 is low-expressed in CRC, once the patients with high expression of lnc-TLCD2-1, the clinical prognosis will be poor.

### Overexpression of lnc-TLCD2-1 Leads to Radiation Resistance

**Figure 3:**
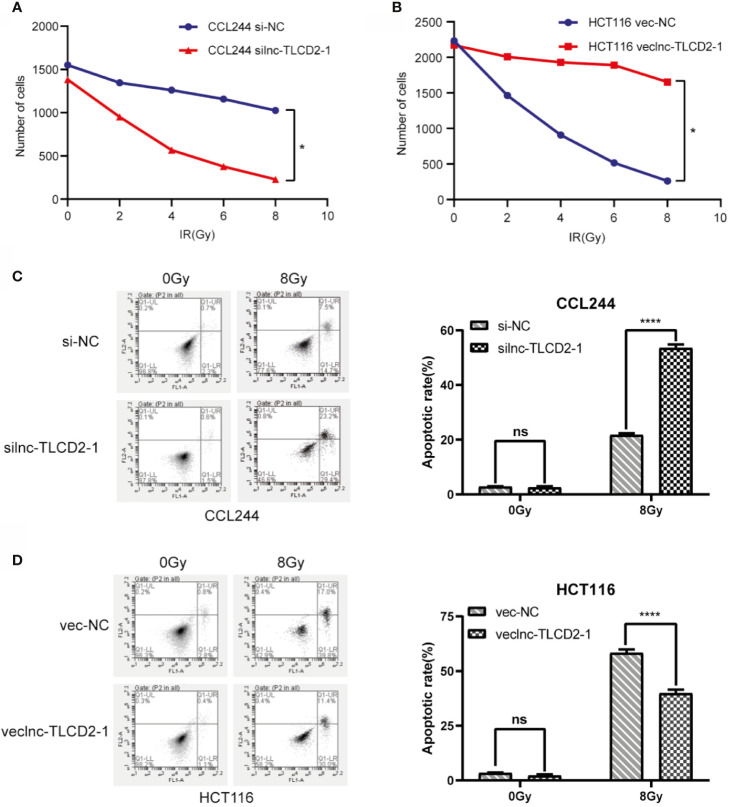
The effect of lnc-TLCD2-1 on radiation resistance of CRC was investigated by cell cloning assay and flow cytometry. **(A)** After inhibition of lnc-TLCD2-1 in CCL244, the number of cells decreased sharply with the increase of radiation intensity. **(B)** After upregulating the expression of lnc-TLCD2-1 in HCT116, the number of cells decreased with the increase of radiation. **(C)** Flow cytometry disclosed that the apoptosis rate of CCL244 with interference of lnc-TLCD2-1 was significantly increased after radiation. **(D)** Flow cytometry revealed that the apoptosis rate of HCT116 with upregulated of lnc-TLCD2-1 was significantly decreased after radiation. (P<=0.05 means statistically significant, ns, no significance, *p < 0.05, ****p < 0.0001.)

Lnc-TLCD2-1 expression was higher in CCL244 than in HCT116, so we down-regulated the expression of lnc-TLCD2-1 in CCL244 by transfection with siRNA, and overexpressed the lnc-TLCD2-1 in HCT116 by plasmid transfection. The results from experiments were surprising. Compared with the negative control group, the number of cell colonies in the CCL244-transfected siRNA group decreased with the increase of radiation dose, but it was obvious that the number of cell colonies decreased more sharply when receiving the same dose of radiation than the negative control group (**Figure 3A**). Compared with the negative control group, the number of cell colonies in the HCT116-transfected veclnc-TLCD2-1 group decreased with the increase of radiation dose (**Figure 3B**), but it was obvious that the number of cell colonies in the latter group decreased more sharply than that in the former group under the same radiation dose. These illustrated the radiosensitivity of both CCL244 and HCT116 was reversed, with CCL244 becoming significantly more sensitive to radiation and HCT116 becoming radiation-resistant. CCL244 was transfected with silnc-TLCD2-1, and the apoptosis rate of cells was significantly increased after 8Gy irradiation compared with negative control (**Figure 3C**). However, after lnc-TLCD2-1 was overexpressed IN HCT116, the apoptosis rate was significantly reduced after irradiation (**Figure 3D**). The above evidence suggested that lnc-TLCD2-1 can cause radiation resistance in CRC.

### lnc-TLCD2-1 Is a Sponge of miR-193a-5p in CRC

**Figure 4:**
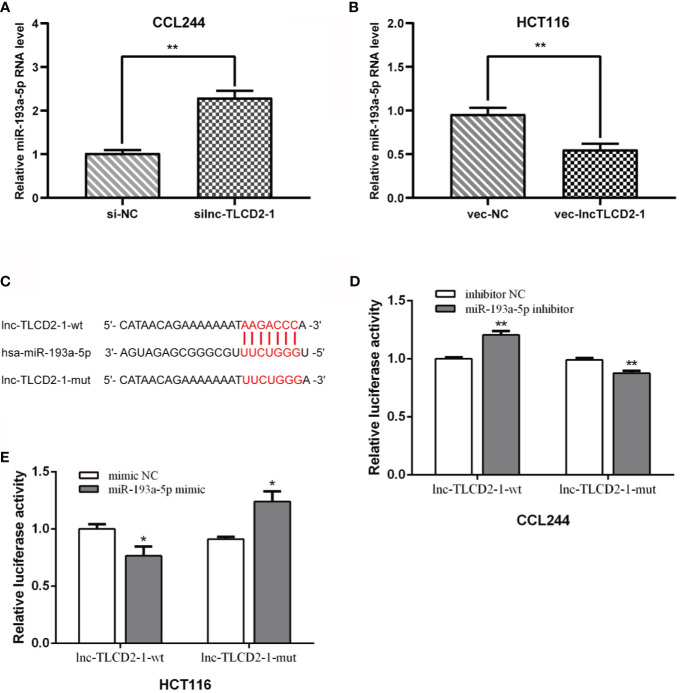
Lnc-TLCD2-1 is a sponge of miR-193a-5p in CRC. **(A)** The expression of miR-193a-5p was increased after CCL244 transfection with silnc-TLCD2-1. **(B)** The expression of miR-193a-5p was decreased after HCT116 transfection with veclnc-TLCD2-1. **(C)** Binding sites of lnc-TLCD2-1 and miR-193a-5p predicted by Starbase, the mutant sites we constructed were demonstrated. **(D)** MiR-193a-5p inhibitor obviously improved the dual luciferase activity of the lnc-TLCD2-1-wt reporter but not lnc-TLCD2-1-mut reporter in CCL244. **(E)** MiR-193a-5p mimic significantly reduced the dual luciferase activity of the lnc-TLCD2-1-wt reporter but not lnc-TLCD2-1-mut reporter in HCT116. (P<=0.05 means statistically significant, *p < 0.05, **p < 0.01).

In order to further explore the mechanism of lnc-TLCD2-1 leading to radiation resistance of CRC, we experimentally verified the results in **Figure 1**. The expression of miR-193a-5p was increased after CCL244 transfection with silnc-TLCD2-1 (**Figure 4A**), while the expression of miR-193a-5p was decreased after HCT116 transfection with veclnc-TLCD2-1 (**Figure 4B**). According to the binding sites of lnc-TLCD2-1 and miR-193a-5p predicted by Starbase (**Figure 4C**), we designed the double luciferase reporter gene plasmids. We found that miR-193a-5p mimic significantly reduced the dual luciferase activity of the lnc-TLCD2-1-wt reporter but not lnc-TLCD2-1-mut reporter in HCT116 (**Figure 4E**). In addition, miR-193a-5p inhibitor obviously improved the dual luciferase activity of the lnc-TLCD2-1-wt reporter but not lnc-TLCD2-1-mut reporter in CCL244 (**Figure 4D**). Based on the above experimental results, we confirmed that lnc-TLCD2-1 directly interacted with miR-193a-5p and negatively regulated its expression in CRC.

### lnc-TLCD2-1 Regulates YY1/NF-кB-P65 by Targeting miR-193a-5p, and Thus May Regulate the Infiltration Environment of CRC Immune Cells

**Figure 5:**
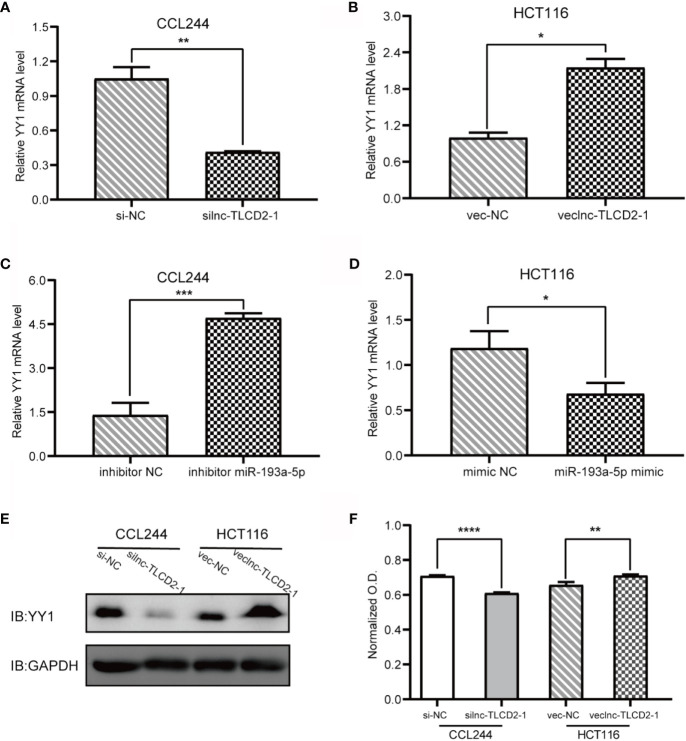
Regulation of lnc-TLCD2-1 and hsa-miR-193a-5p on YY1, and the effect of lnc-TLCD2-1 on CRC proliferation. **(A)** In CCL244, the expression of YY1 decreased after interference of lnc-TLCD2-1. **(B)** The expression of YY1 increased after overexpression of lnc-TLCD2-1 in HCT116. **(C)** YY1 was increased after inhibition of miR-193a-5p in CCL244. **(D)** YY1 was down-regulated after upregulation of miR-193a-5p in HCT116. **(E)** Western Blot detected the change of YY1 in protein level. **(F)** CRC proliferation was inhibited or promoted by interfering or upregulating the expression of lnc-TLCD2-1 in CCL244 and HCT116, respectively. (P<=0.05 means statistically significant, *p < 0.05, **p < 0.01, ***p < 0.001, ****p < 0.0001.)

**Figure 6:**
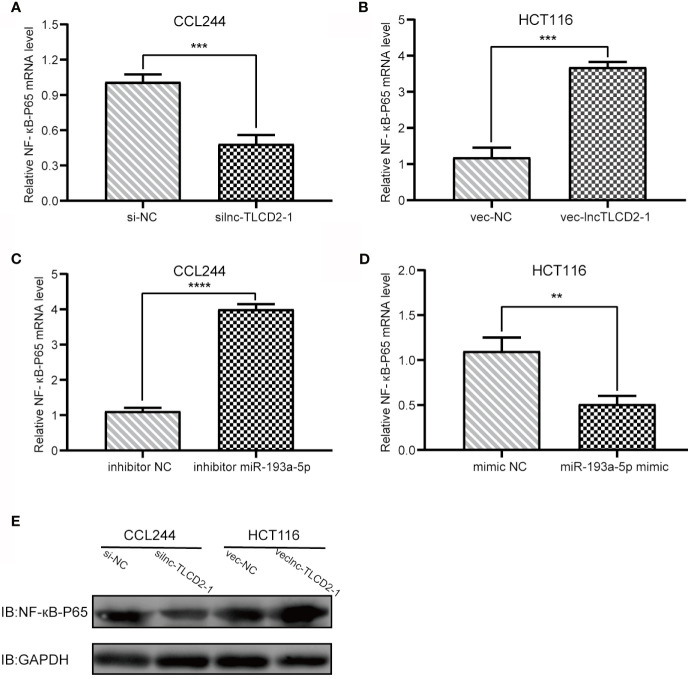
Regulation of lnc-TLCD2-1 and hsa-miR-193a-5p on NF-кB-P65. **(A)** In CCL244, the expression of NF-кB-P65 decreased after interference of lnc-TLCD2-1. **(B)** The expression of NF-кB-P65 increased after overexpression of lnc-TLCD2-1 in HCT116. **(C)** NF-кB-P65 was increased after inhibition of miR-193a-5p in CCL244. **(D)** NF-кB-P65 was down-regulated after upregulation of miR-193a-5p in HCT116. **(E)** Western Blot detected the change of NF-кB-P65 in protein level. (P<=0.05 means statistically significant, **p < 0.01, ***p < 0.001, ****p < 0.0001.)

**Figure 7:**
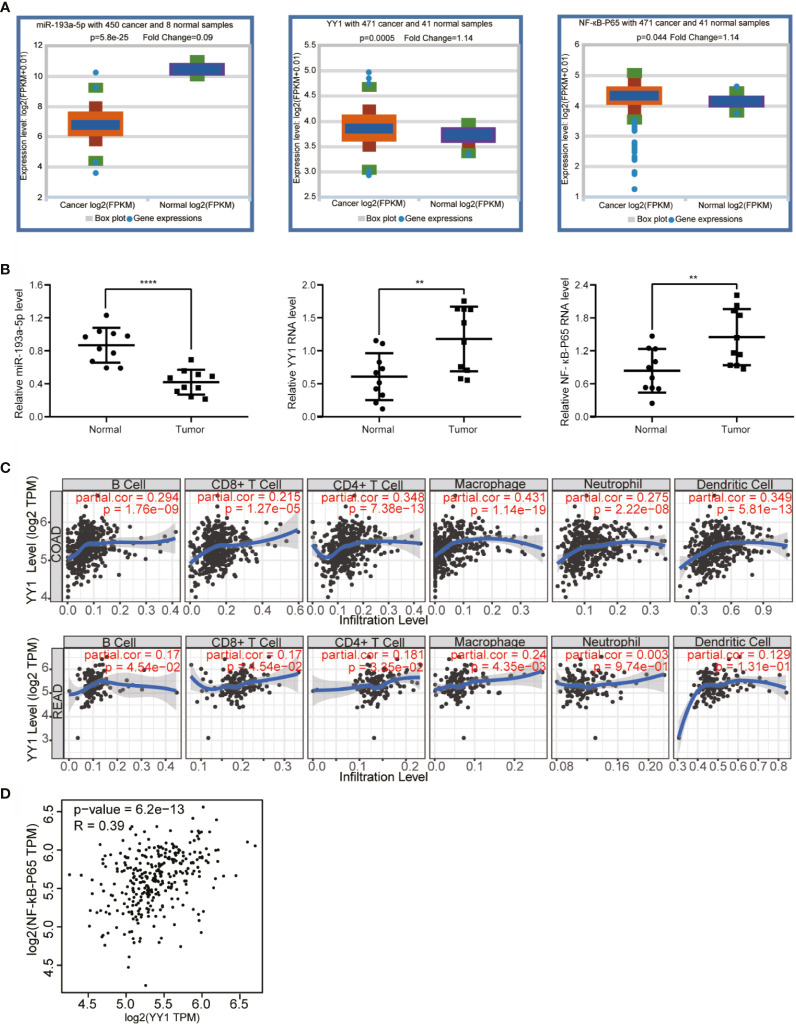
Bioinformatics analysis and clinical sample verification. **(A)** Starbase demonstrated that miR-193a-5p is dramatically downregulated in CRC, while YY1 and NF-кB-p65 were upregulated in CRC compared with normal colorectal tissues. **(B)** The expression levels of miR-193a-5p, YY1 and NF-кB-p65 in CRC tissues were verified in 10 paired samples by qRT-PCR. **(C)** The effect of YY1 on the immune cell infiltration of CRC was investigated by TIMER (COAD stands for colon cancer, READ stands for rectal cancer). **(D)** The correlation between YY1 and NF-кB-p65 was explored by GEPIA. (P<=0.05 means statistically significant, **p < 0.01, ****p < 0.0001.)

As mentioned above, we have proved the direct interaction between lnc-TLCD2-1 and hsa-miR-193a-5p, and according to previous reports, hsa-miR-193a-5p regulates the proliferation and metastasis of CRC by interfering with YY1. Meanwhile, it has been reported that NF-кB-P65 and YY1 can form complexes to regulate the transcription of downstream genes (24–27). Therefore, we detected the changes of YY1 and NF-кB-P65 at mRNA level after CCL244 transfection with silnc-TLCD2-1 and HCT116 transfection with veclnc-TLCD2-1. As expected, the mRNA levels of YY1 and NF-кB-P65 decreased in CCL244 (**Figures 5A**, **6A**), whereas the opposite was true in HCT116 (**Figures 5B**, **6B**). For further verification, such changes in YY1 and NF-кB-P65 were indirectly caused by the action of lnc-TLCD2-1 on hsa-miR-193a-5p, we then transfected CCL244 with hsa-miR-193a-5p inhibitor and HCT116 with hsa-miR-193a-5p mimic. As a result, hsa-miR-193a-5p can obviously inhibit the expression of YY1 and NF-кB-P65 in colorectal cancer cells (**Figures 5C, D**, **6C, D**). Both of them were up-regulated by lnc-TLCD2-1 at the protein level (**Figures 5E**, **6E**). **Figure 5F** disclosed that lnc-TLCD2-1 can promote CRC cell proliferation. Starbase database analyzed the expression of miR-193a-5p,YY1 and NF-кB-P65, and the results showed that the first molecule was low expressed in CRC, while the latter two were both high expressed (**Figure 7A**). And the results were validated using 10 pairs of clinical samples (**Figure 7B**). GEPIA showed that the expression of YY1 and NF-кB-P65 in CRC was moderately correlated, which also confirmed that they could form complexes (**Figure 7D**). The expression of YY1 is significantly correlated with tumor microenvironment infiltration of B cells, CD8+T cells, CD4+T cells, macrophages, neutrophils and dendritic cells, the six immune cells in colorectal cancer (**Figure 7C**). This suggests that lnc-TLCD2-1 may affect the immune microenvironment of CRC by regulating the expression of YY1, and further affect the clinical prognosis of patients. As mentioned above, we can make it clear that lnc-TLCD2-1 regulates the expression of YY1/NF-кB-P65 by directly targeting hsa-miR-193a-5p.

### GO and KEGG Enrichment Analysis of Similar Genes Co-Expressing With lnc-TLCD2-1 in CRC

**Figure 8:**
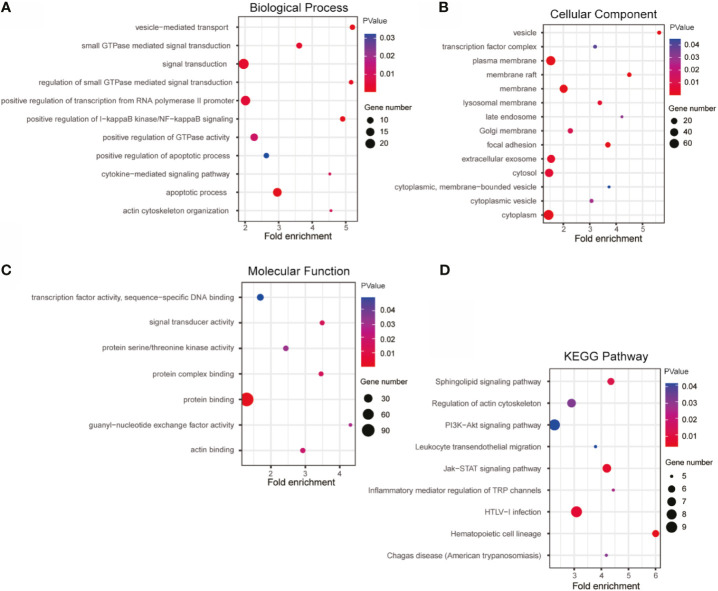
GO and KEGG enrichment of lnc-TLCD2-1 with its co-expression genes. **(A)** Biological Process was mainly enriched in signal transduction, positive regulation of transcription from RNA polymerase II promoter and apoptotic process. **(B)** Cellular Component was mainly enriched in plasma membrane, membrane, extracellular exosome, cytosol and cytoplasm. **(C)** Molecular Function analysis indicated that most of co-expression genes were enriched in protein binding. **(D)** KEGG pathway analysis revealed that HTLV-I infection, PI3K-Akt signaling pathway and Jak-STAT signaling pathway were mainly enriched. (p value<=0.05, Gene number>=5).

Based on Pearson Correlation Coefficient (PCC)>=0.5 as the selection criteria, GO and KEGG enrichment analysis was performed on the 190 similar genes with co-expression relationship with lnc-TLCD2-1, detailed results of enrichment analysis were given in **Supplemental Tables 3**–**6**. Biological Process analysis showed that lnc-TLCD2-1 co-expressed genes were obviously associated with signal transduction, positive regulation of transcription from RNA polymerase II promoter, positive regulation of I-kappaB kinase/NF-kappaB signaling, apoptotic process, small GTPase mediated signal transduction (**Figure 8A**); Cellular Component analysis showed that the genes were mainly enriched in cytoplasm, cytosol, plasma membrane, extracellular exosome (**Figure 8B**); Molecular Function enrichment analysis showed that this group genes mainly functions in protein binding, protein complex binding, signal transducer activity and so on (**Figure 8C**). As for enrichment in the KEGG pathway, mainly in the following: HTLV-I infection, Jak-STAT signaling pathway, regulation of actin cytoskeleton, sphingolipid signaling pathway, hematopoietic cell lineage, inflammatory mediator regulation of TRP channels, chagas disease (American trypanosomiasis), leukocyte transendothelial migration, PI3K-Akt signaling pathway (**Figure 8D**). What is particularly noteworthy in the above results is that lnc-TLCD2-1 is significantly enriched in the positive regulation of I-kappaB kinase/NF-kappaB signaling, which further confirms the regulation of YY1/NF-кB-P65 by lnc-TLCD2-1.

## Discussion

Colorectal cancer (CRC) is the third most common cancer in the world and accounts for a significant proportion of cancer-related deaths. At the same time, radiation resistance has always been a difficult problem in CRC treatment, especially advanced CRC, and radiation resistance profoundly affects the clinical prognosis of patients with CRC. Therefore, it is of great significance to study the mechanism of radiation resistance in CRC to improve the prognosis of patients. Recent studies have shown that IDO1 inhibition can enhance the radiotherapy effect of CRC (28), miR-145 is a novel therapeutic target for CRC to overcome radiation resistance (29), JAK2/STAT3/CCND2 axis and COASY/PI3K signal mediate radiation resistance of CRC (30, 31). However, little is known about the role of lncRNA in regulating radiosensitivity of CRC. Previous studies on lncRNA mainly focused on the effects of the interaction between lncRNA and microRNA on the proliferation, cell cycle and invasion ability of cancer cells.

To our knowledge, lnc-TLCD2-1 is a recently discovered lncRNA, very little is known about lnc-TLCD2-1, especially in CRC. Our study found significant differences in lnc-TLCD2-1 expression levels between radiation-resistant CCL244 and radiation-sensitive HCT116. After CCL244 was irradiated, the expression of lnc-TLCD2-1 was significantly decreased, while that of HCT116 was significantly increased. It is suggested that lnc-TLCD2-1 may have an important correlation with the radiation sensitivity of CRC. Considering that the basic expression of lnc-TLCD2-1 in CCL244 is much higher than that in HCT116, we then reduced its expression in CCL244 and upregulated it in HCT116, and conducted a comparative experiment. Subsequently, we conducted cell coloning experiments and flow cytometry apoptosis experiments, and it was found that after the overexpression of lnc-TLCD2-1, cell apoptosis was reduced; on the contrary, after the decrease of lnc-TLCD2-1, cell apoptosis was increased. This indicates that lnc-TLCD2-1 can cause radiation resistance of CRC. It seems paradoxical that the level of lnc-TLCD2-1 in radiation-resistant CCL244 is decreased after radiation exposure. We believe that after receiving radiation, there are other regulatory pathways in CRC cells that have an impact on the expression of lnc-TLCD2-1. Our study was conducted to identify the lnc-TLCD2-1 effect on the CRC radiosensitivity, as for the regulation of other pathways is the target of our further research in the future.

At the same time, bioinformatics analysis of lnc-TLCD2-1 was performed to explore the internal mechanism of lnc-TLCD2-1 leading to radiation resistance in CRC. The expression level of lnc-TLCD2-1 in CRC tissues was significantly lower than that in normal tissues. In addition, high expression of lnc-TLCD2-1 predicted shorter Disease Specific Survival and Overall Survival in patients. This might confirm that CRC radiation resistance closer correlations with lnc-TLCD2-1, because patients with radiation resistance will have a worse prognosis and shorter survival.

A growing body of research points to intricate interactions between different RNAs, including protein-coding mRNAs and non-coding RNAs, such as long non-coding RNAs, microRNAs, and circular RNAs. Among them, studies have discovered that lncRNAs can function as endogenous miRNA sponges, suggests that lncRNAs compete with mRNAs for miRNA binding and thus regulate downstream target genes expression. This regulatory network between RNAs has a major impact on the development of disease in humans (32). For example, lncRNA PVT1 regulates hypertrophy through miR-214-mediated expression of TFR1 and TP53 (33). LncRNA SND1-IT1 accelerates proliferative and migratory abilities of osteosarcoma *via* sponging miRNA-665 to upregulate POU2F1 (34), further affect the clinical prognosis of patients with osteosarcoma. LncRNA MIR497HG targets the miRNA-128-3p/SIRT1 axis to inhibit the proliferation and migration of retinal endothelial cells under high glucose treatment (35). LncRNA CDKN2BAS predicts a poor prognosis in patients with HCC and promotes its metastasis through miR-153-5p/ARHGAP18 signaling axis (36).

We predicted miRNAs interacting with lnc-TLCD2-1 using bioinformatics databases. We were surprised to find that the database predicted a target region between hsa-miR-193a-5p and lnc-TLCD2-1, and at the same time, hsa-miR-193a was significantly decreased in irradiated HCT116 and increased in CCL244.This leads us to speculate that lnc-TLCD2-1 plays a negative regulatory role on hsa-miR-193a. The dual luciferase reporter gene confirmed this prediction. The analysis of gene expression detected by qRT-PCR indicated that lncTLCD2-1 indeed had a negative regulation relationship with miR-193a-5p. The expression of miR-193a-5p was induced after the transfection of lnc-TLCD2-1 siRNA. The expression level of miR-193a-5p was negatively affected after transfection of the overexpression vector of lnc-TLCD2-1.

Transcription factors regulate gene expression, and more and more reports prove that transcription factors play a promoting or inhibiting role in the occurrence and development of cancer or other diseases (37–40). As a common and important transcription factor in mammals (41), YY1 interact with guanine quadruplets to regulate DNA looping and gene expression (42). According to previous literature reports, it has been confirmed that miR-193a-5p down-regulated YY1. According to our experimental results, it was proved that lnc-TLCD2-1 could indirectly promote the expression of YY1 in CRC by targeting miR-193a-5p. In addition, some studies have confirmed that YY1 can promote the proliferation of CRC, and our experimental results demonstrated that lnc-TLCD2-1 can promote the proliferation of CRC. Our study found that the lnc-TLCD2-1/miR-193a-5p axis can regulate NF-кB -p65, which means that lnc-TLCD2-1 regulates YY1/NF-кB -p65 complex by targeting miR-193a-5p, thus regulating the transcription of other genes. Therefore, the effect of lnc-TLCD2-1 on the radiosensitivity of CRC is of far-reaching significance, and its related molecular signaling pathway mechanism may be extremely complex, which needs further exploration in the future. Bioinformatics analysis showed that miR-193a-5p was low expressed in colon cancer, while YY1 and NF-кB -p65 were overexpressed, the results were validated using 10 pairs of clinical samples, and the latter two were moderately correlated in CRC (Pearson, R=0.39), in addition to these, enrichment analysis shows that lnc-TLCD2-1 is associated with positive regulation of NF-KappaB signaling. These are also consistent with our research results. Finally, bioinformatics analysis revealed a significant correlation between YY1 expression and immune cell infiltration in colorectal cancer. It is suggested that lnc-TLCD2-1 may target YY1/NF-кB -p65 axis by miR-193a-5p-targeting to regulate the environment of tumor immunoinvasion in colorectal cancer, then modulate the radiosensitivity of CRC and affect the clinical prognosis of CRC patients.

## Conclusion

In conclusion, lnc-TLCD2-1 can induce radiation resistance of CRC, and the possible mechanism is the regulation of YY1/NF-кB -p65 by targeting miR-193a-5p.Lnc-TLCD2-1 promote the proliferation of CRC, and patients with high expression of it have a shorter survival. Our study provides a new potential target for the treatment of CRC, especially radiation-resistant CRC, and lnc-TLCD2-1 can be used as a potential biomarker for prognosis of CRC patients.

## Data Availability Statement

The original contributions presented in the study are included in the article/**Supplementary Material**. Further inquiries can be directed to the corresponding authors.

## Author Contributions

QY and WZ completed the main writing, experiments, and bioinformatics analysis. XZ and WS completed part of the experiments and writing, CX and XY completed the design and guidance of the subject, and made major revisions to the manuscript. All authors contributed to the article and approved the submitted version.

## Funding

This project was supported by the following funds: Jiangsu Postgraduate Research Innovation Program (Grant No. KYCX21_2979), Jiangsu Provincial Health Commission Scientific Research Project (Grant No. QNRC2016873 and No. CXTD-16), the Subject of Suzhou Science and Technology Bureau (Grant No. SYS2018055), and the Gusu Health Talents Project (Grant No. GSWS2020037).

## Conflict of Interest

The authors declare that the research was conducted in the absence of any commercial or financial relationships that could be construed as a potential conflict of interest.

## Supplementary Material

Supplemental Table 1 Differential RNA of CCL244.

Supplemental Table 2 Differential RNA of HCT116.

Supplemental Table 3 Biological Process enrichment of coexpressed genes.

Supplemental Table 4 Cellular Component enrichment of coexpressed genes

Supplemental Table 5 Molecular Function enrichment of coexpressed genes.

Supplemental Table 6 KEGG pathway analysis of coexpressed genes.

Supplementary Data Sheet 1 The sequence of lnc-TLCD2-1.
